# Rolling Circle Amplification on a Bead: Improving the Detection Time for a Magnetic Bioassay

**DOI:** 10.1021/acsomega.2c07992

**Published:** 2023-01-18

**Authors:** Darío Sánchez Martín, Reinier Oropesa-Nuñez, Teresa Zardán Gómez de la Torre

**Affiliations:** † Department of Material Sciences and Engineering, Division of Nanotechnology and Functional Materials, Ångström Laboratory, Uppsala University, Box 534, SE-751 21 Uppsala, Sweden; ‡ Department of Material Sciences and Engineering, Division of Solid-State Physics, Ångström Laboratory, Uppsala University, Box 534, SE-751 21 Uppsala, Sweden

## Abstract

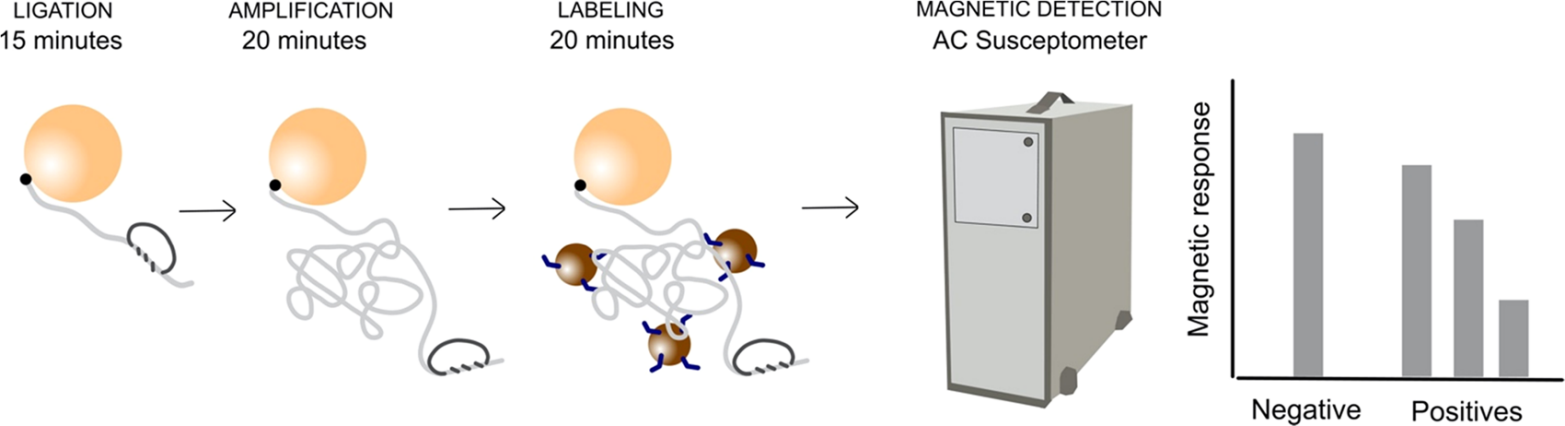

Detection of pathogens has become increasingly important, especially in the face of outbreaks and epidemics all over the world. Nucleic acid detection techniques provide a solid base to detect and identify pathogens. In recent years, magnetic sensors and magnetic labels have become of more interest due to their simplicity of use, low cost, and versatility. In this work, we have used the isothermal DNA amplification technique of rolling circle amplification (RCA) in combination with oligo-functionalized magnetic nanoparticles. Detection of RCA products takes place through specific binding between magnetic nanoparticles and RCA products. Upon binding, the relaxation frequency of the nanoparticle changes. This change was measured using an AC susceptometer. We showcase that the RCA time can be reduced for a quicker assay when performing the RCA on the surface of micrometer-sized beads, which consequently increases the hydrodynamic volume of the RCA products. This, in turn, increases the Brownian relaxation frequency shift of the nanoparticles upon binding. We performed optimization work to determine the ideal quantity of micrometer-sized particles, oligo-functionalized nanoparticles, and the amplification time of the RCA. We show that the detection of 0.75 fmol of preamplification synthetic target is possible with only 20 min of amplification time. Finally, we showcase the high specificity of the assay, as the functionalized nanoparticles are unable to bind to amplified DNA that does not match their labels. Overall, this paves the way for a simple bioassay that can be used without expensive laboratory equipment for detection of pathogens in outbreak settings and clinics around the world.

## Introduction

Viral and bacterial outbreaks have showcased the need for easy-to-use assays to detect the pathogen responsible for the outbreaks, as well as its characteristics. The presence of rapid diagnostic tests can reduce the size of the outbreaks.^1^ Nucleic acid detection is a strong option as, encoded in the nucleic acids, we can find information about bacterial species, antibiotic resistance among them, the presence or absence of plasmids, or in the case of virus, the type and strain.^2,3^ However, common laboratory techniques may be hard to use in the case of outbreaks, and instead, simpler assays are preferred. This is especially true in low- and middle-income countries, where laboratory testing has been neglected and is not as commonplace as in higher income countries.^4^

There is a growing interest in analytical techniques based on magnetic labels because of their physical and chemical stability, inexpensive methods of production, and ability to functionalize them with different types of biomolecules. Also, there is no significant magnetic background present in most samples of interest, which makes detection and magnetic manipulation with magnetic nanoparticles easier without affecting the biomolecular interactions.^5^ Several different types of magnetic biosensors have been developed during the past decades, such as giant magnetoresistance and planar Hall effect sensors, superconducting quantum interference devices, and fluxgate magnetometers.^6,7^ These detection techniques can be categorized as either surface-based or volumetric-based sensors. Surface-based sensors offer high sensitivity, allowing detection of a single magnetic particle. However, these techniques require laborious sample and substrate preparation. On the other hand, volumetric-based sensors provide simpler and rapid sample preparation and detection. The Brownian relaxation principle is an example of a volumetric-based method and has been used in biosensing for a wide range of applications.^8,9^ Particles exhibiting Brownian relaxation behavior are functionalized with probe molecules, and when these molecules bind to a target molecule, it causes an increase in the hydrodynamic size of the nanoparticle. The frequency at which the nanoparticle relax is inversely proportional to its hydrodynamic size, and an increase in size (e.g., binding to a target) results in a decrease of the relaxation frequency.^8,10^ This size increase is detected as a peak shift to lower frequencies in the imaginary part (χ″) of the complex susceptibility spectrum (χ = χ – *i*χ″). Alternatively, the presence of target molecules can be monitored by measuring the amplitude decrease of the Brownian relaxation peak of the remaining unbound nanoparticles upon target binding.^11,12^ The bioassay in this work uses this strategy in combination with the rolling circle amplification (RCA) method to achieve a massively enhanced response upon binding of the magnetic nanoparticles to RCA products (RCPs). The RCPs are produced in a series of reactions, starting with DNA target recognition using the padlock probe technology.^13,14^ Padlock probes are oligonucleotides where both ends are designed to exactly match the target sequence. The ends of the probe can be joined by DNA ligase, and the probe molecule are thereby transformed into a DNA circle that can subsequently act as templates for RCA, which is a method for linear polymerization creating long single-stranded DNA products.^15,16^

Earlier studies describing magnetic nanoparticle-based bioassays combined with RCA showed that it was needed to have long RCA times (at least 60 min) to achieve a low limit of detection (LOD).^12,17−19^ In this study, we explore the possibility of performing RCA on the surface of 1 μm magnetic particles (MyOne streptavidin T1 Dynabeads) to maintain the massive size of the target while decreasing the RCA time, hence reducing the assay time. We investigate how different parameters such as the Dynabead concentration, nanoparticle concentration, and RCA time affect the bioassay system in terms of sensitivity. Finally, the specificity of the assay was tested with different concentrations of noncomplementary RCPs.

## Results and Discussion

### Varying the Dynabead Concentration

**Figure 1:**
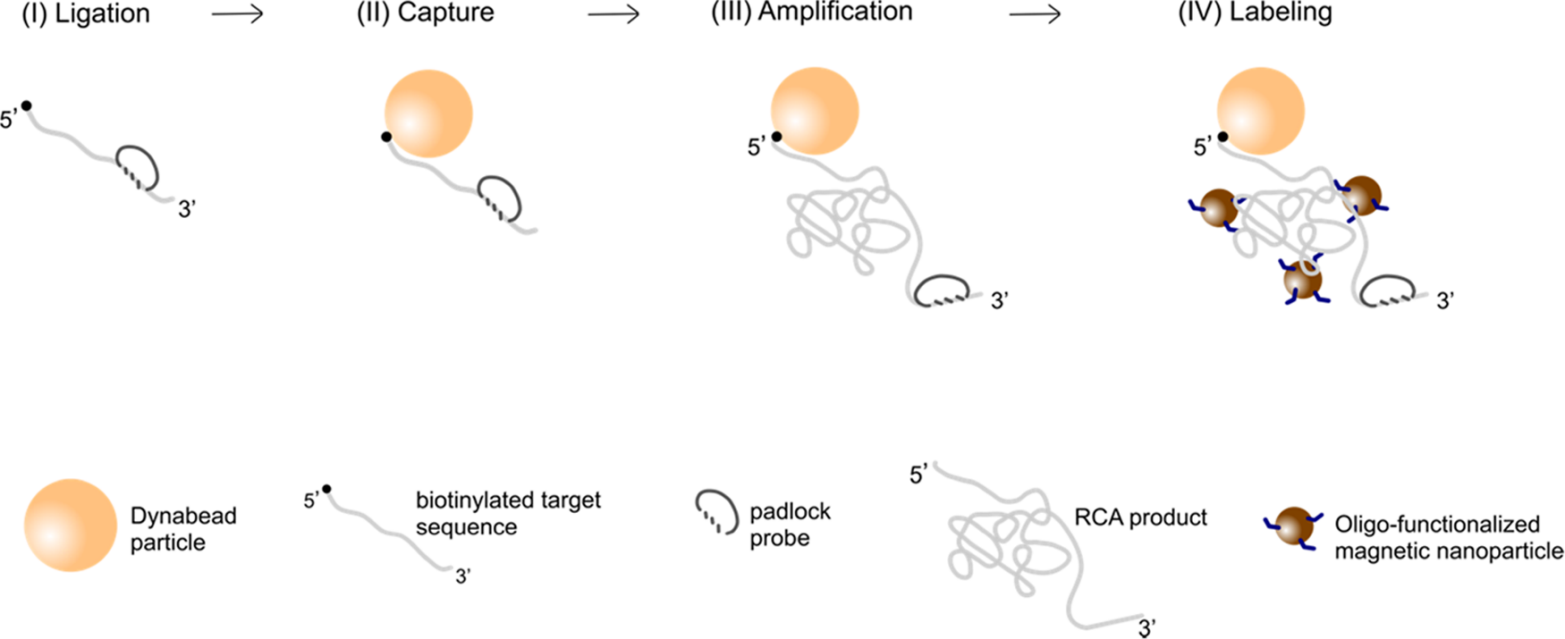
Overview of the molecular reactions and the magnetic labeling used in the present bioassay. The target recognition and ligation are performed using the padlock probe technology (I). RCA is performed on the surface of microbeads (Dynabeads) after a capturing step of the padlock probe–target complex (II, III). The RCA products are magnetically labeled using oligonucleotide-functionalized 100 nm nanoparticles (IV).

The principle behind the proposed bioassay is presented in Figure 1. In summary, the RCPs are produced in a series of reactions, starting with target recognition using the padlock probe technology. The synthetic targets are tagged with biotin groups at the 5′-end of the sequences making the probe–target complex able to bind to the surface of the Dynabeads. The reacted probes constitute suitable templates for RCA creating long single-stranded DNA products. The presence of the RCPs is monitored by hybridization of oligonucleotide-functionalized magnetic nanoparticles to the RCPs, increasing drastically the hydrodynamic volume of the nanoparticles. The action of the Dynabeads is twofold. First, they can isolate the bound targets in a washing step from an unwanted material in the sample. Second, the hydrodynamic volume change that the magnetic nanoparticles suffer upon binding to the RCPs is enhanced by the fact that the RCPs are linked to a T1 Dynabead of 1 μm diameter, much larger than the 100 nm diameter of the magnetic nanoparticles.

**Figure 2:**
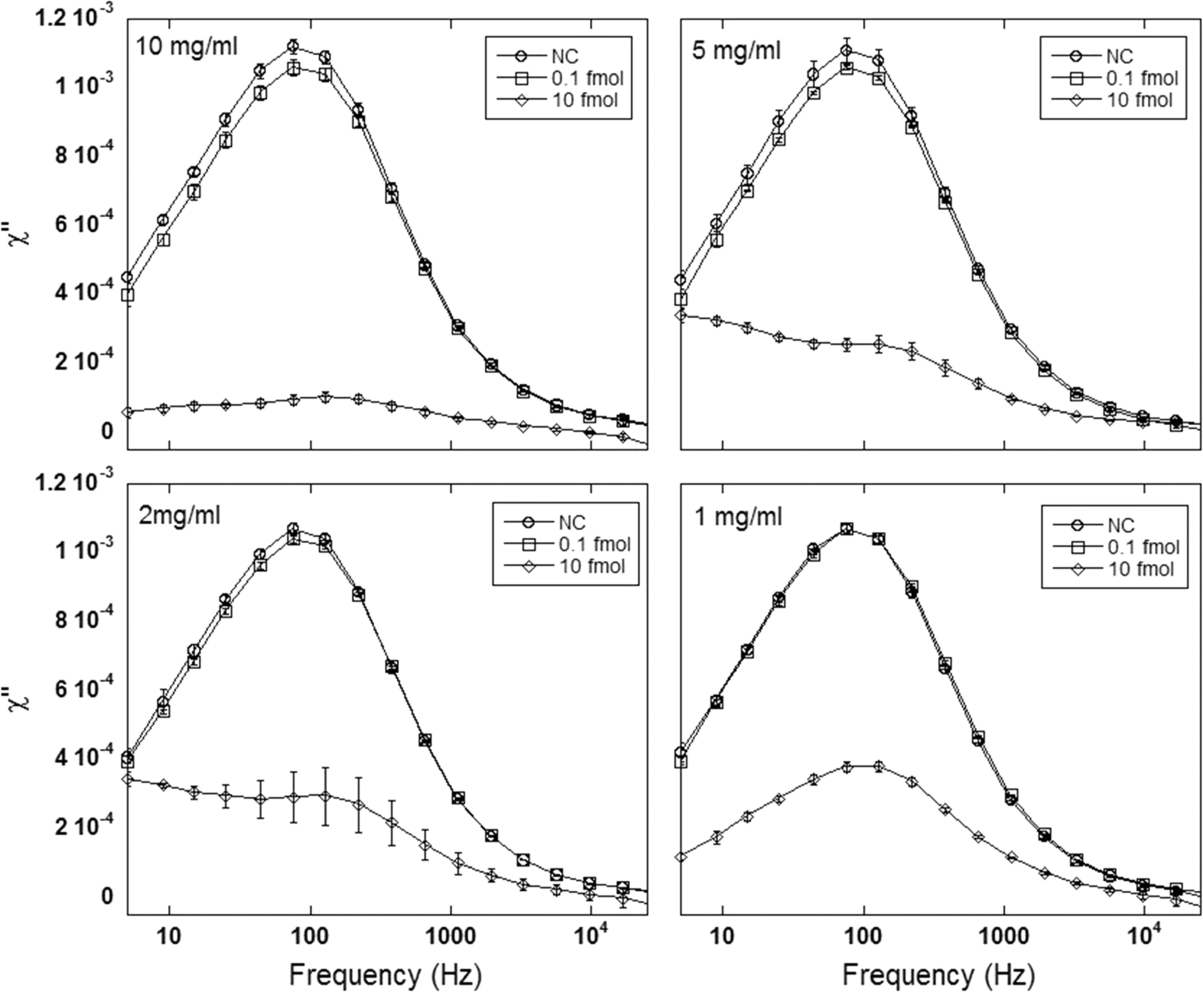
Imaginary part of the complex susceptibility spectra of two target DNA concentrations (0.1 and 10 fmol) and their respective negative controls at different Dynabead concentrations (1, 2, 5, and 10 mg/mL). The RCPs were amplified for 60 min. The error bars indicate the standard deviation of three independent replicates.

To investigate the influence of Dynabead concentration in the assay performance, four different concentrations were tested (1, 2, 5, and 10 mg/mL). Figure 2 shows the imaginary part of the complex susceptibility spectra (χ″) for different Dynabead concentrations and two amounts of target DNA (0.1 and 10 fmol) and their respective NC. Here, 2 mg/mL magnetic nanoparticles were used. From the figure, one can see that the peak amplitude for the 0.1 and 10 fmol samples decreases with increasing Dynabead concentration, and this trend is more prominent for the highest DNA concentration where the peak decrease for 10 mg/mL Dynabeads is about 90% compared to about 60% for the case of 1 mg/mL. The large peak reduction and the flat shape of the magnetization curve for the 10 fmol DNA sample indicates that almost all nanoparticles have been bound to the RCPs (see Figure S1). For 5 and 2 mg/mL Dynabeads and 10 fmol of target DNA, there are two peaks overlapping where the low-frequency peak corresponds to the magnetic nanoparticles hybridized to the RCPs and the high-frequency peak corresponds to free nanoparticles. For the samples containing 1 mg/mL Dynabeads and 10 fmol of DNA target, the peak centered at 75 Hz, corresponding to unbound nanoparticles, shows a reduction of about 60% compared to the negative control (NC). These results indicate the capture of almost all targets when using a high Dynabead concentration, such as 10 mg/mL, resulting in a higher number of RCPs in the sample compared to samples containing 1 mg/mL Dynabeads.

To investigate if the magnetic response of the Dynabeads would add to the magnetization background of magnetic nanoparticles, we measured on an NC sample containing only Dynabeads (Figure S1). From the graph, one can see that the curve is completely flat and thus does not contribute to the background signal of the magnetic nanoparticles.

### Varying the Nanoparticle Concentration

**Figure 3:**
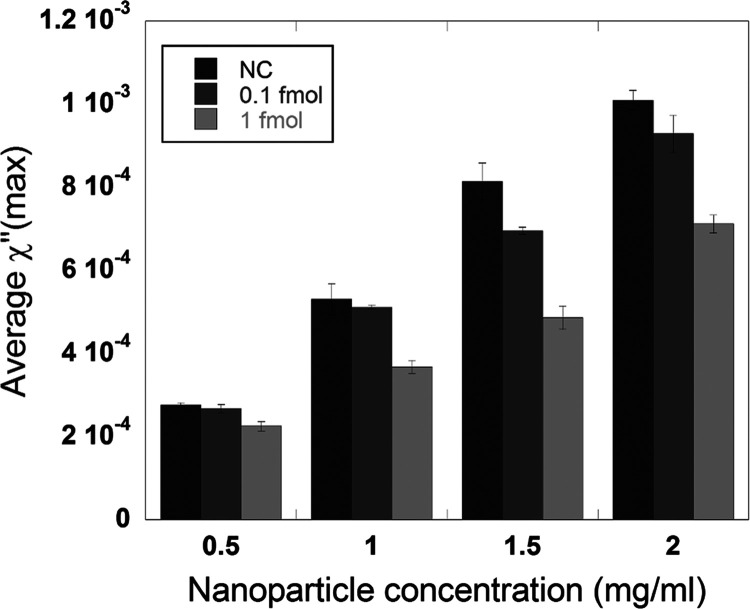
Mean maximum χ″-peak value of two DNA target concentrations (0.1 and 1 fmol) and their respective negative controls at different nanoparticle concentrations (0.5, 1, 1.5, and 2 mg/mL). The RCPs were amplified for 60 min. The error bars indicate the standard deviation of three independent replicates.

The results of varying the nanoparticle concentration (number of magnetic labels) are presented in Figure 3. The imaginary parts of the complex susceptibility spectra for the different samples are presented in Figure S2. Here, a concentration of 10 mg/mL Dynabead particles and an RCA time of 60 min were used. The mean maximum χ″-peak value for the NC, 0.1 fmol, and 1 fmol samples decreases almost linearly with decreasing nanoparticle concentration (*R*^2^ = 0.995, 0.997, and 0.979 for the NC, 0.1 fmol, and 1 fmol samples, respectively). *R*^2^ represents how much of the variability is explained by the linear model, with 100% being *R*^2^ equal to 1. However, there is a slightly larger difference between the positive samples and their corresponding NC sample (Δχ″ = χ″(NC) – χ″(target concentration)) in the case of 1.5 mg/mL nanoparticles. This nanoparticle concentration was therefore selected to be used in the subsequent experiments.

### Varying the RCA Time

**Figure 4:**
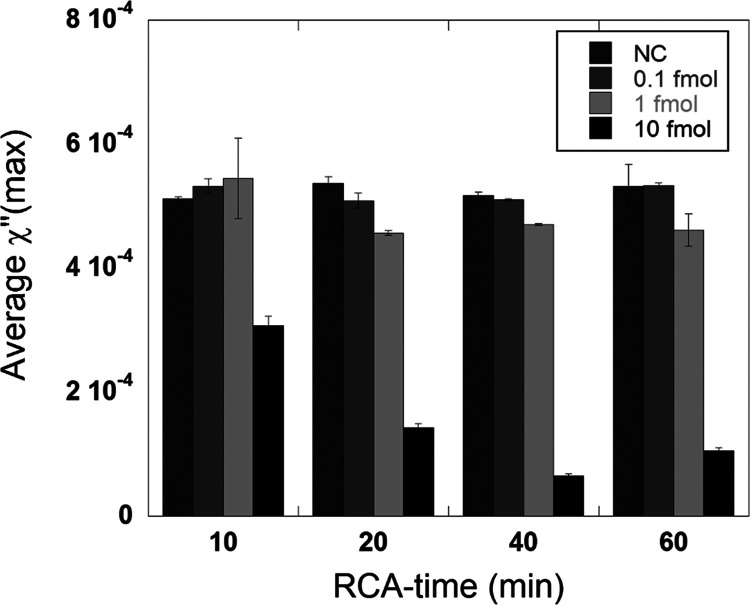
Mean maximum χ″-peak value of three target DNA amounts (0.1, 1, and 10 fmol) and their respective negative controls for different RCA times (10, 20, 40, and 60 min). Magnetic nanoparticles at a concentration of 1.5 mg/mL functionalized detection oligos were used. The error bars indicate the standard deviation of three independent replicates.

The hybridization efficiency by varying the RCA time was investigated, and the results are presented in Figure 4. Four RCA times (10, 20, 40, and 60 min) were evaluated, and for each RCA time, three target DNA amounts were analyzed. The results show that the mean maximum χ″-peak value for the highest DNA target amount (10 fmol) for both 60 and 40 min is very low compared to the NC, and its corresponding curves are almost flat (Figure S3). There is a peak decrease of 87 and 80% for the 60 and 40 min samples, respectively, indicating that a large amount of the nanoparticles is hybridized to the RCPs. A decrease in the peak amplitude was found for the 20 and 10 min samples where the peak intensity decreased with 73 and 40%, respectively. For the 1 fmol DNA target samples, the reduction in peak amplitude was calculated to be 13, 9, 15, and 0% for 60, 40, 20, and 10 min, respectively. These results show that the hybridization efficiency for 60, 40, and 20 min is almost the same for this particular target DNA amount, but there was no detectable hybridization for 10 min. For the lowest target DNA amount (0.1 fmol), a small decrease is observed for 60, 40, and 20 min, whereas for 10 min, there is no detectable hybridization between nanoparticles and RCPs. It should be noted that, even if there is a small difference between the NC samples and the 0.1 fmol samples (for 20, 40, and 60 min), there is no significant difference between the samples based on the LOD criteria (three standard deviations from the NC sample mean).

### Dose–Response Curve

Quantitative detection of target DNA by the proposed and optimized detection method was investigated. Different amounts of synthetic target DNA, ranging from 0.5 to 50 fmol, were reacted and amplified through a padlock probe and the RCA technique, respectively. The RCA was conducted for 20 min on the surface of Dynabeads (10 mg/mL), and RCPs were subsequently labeled with 1.5 mg/mL probe-tagged nanoparticles.

**Figure 5:**
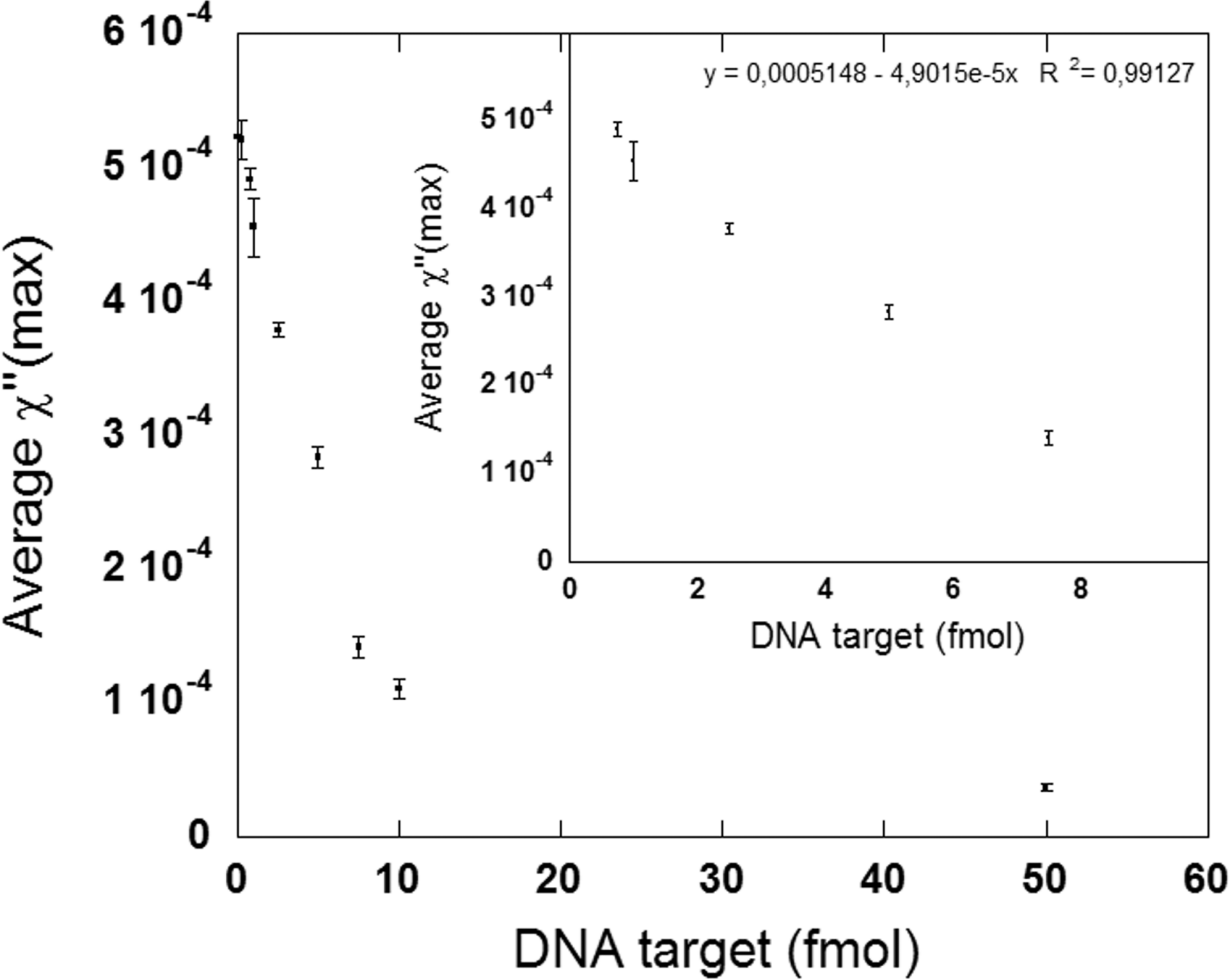
Dose–response curve for the proposed bioassay. The inset shows the dose–response curve for concentrations between 0 and 10 fmol. In the assay, 10 and 1.5 mg/mL Dynabead and nanoparticles were used, respectively. The RCA was conducted for 20 min. The error bars indicate the standard deviation of three independent replicates.

The dose–response curve is shown in Figure 5, and the full imaginary part of the complex susceptibility spectra for the different samples are presented in Figure S4. It can be seen that the mean χ″-peak value decreases with an increasing amount of target DNA. A linear correlation between the peak amplitude and DNA amount was obtained between 0.75 fmol and 7.5 fmol with a mean coefficient of variation (CV) of 4.3%. An LOD of 0.75 fmol was obtained using the proposed bioassay. The LOD is calculated as the average values for the NC minus 3 times the SD. This is also described in the Materials and Methods section.

Table 1 presents different DNA detection methods based on RCA. The LOD of our assay is 0.75 fmol, which is less sensitive than the other presented bioassays. Despite the fact that our method is less sensitive, the main advantage is that it allows for much faster detection results. All other methods have an assay time that runs for hours, whereas the presented method give results under 1 h. One of the assays has also shown nonspecific binding of the particles to the RCPs, which has not been observed in this study.

**Table 1:** Comparison of Different RCA-Based Assays

amplification method	readout method	LOD	assay time	reference
RCA	magnetic	0.75 fmol	55 min	this work
RCA	visual, spectroscopy, and magnetic	0.4 fmol	less than 2 h	(20)
RCA	electrochemical	3 amol	6 h	(21)
RCA	visual (magnetic particles)	0.6 fmol	6 h	(22)
RCA	colorimetric (phenol red)	3 pM	3 h	(23)

RCA is a technique that provides linear amplification. Other amplification techniques that offer a higher amount of amplification products could be used to increase the sensitivity. Other RCA-based methods, such as C2CA, HRCA, or BRCA, could be employed in a similar fashion.

### Specificity of the Assay

**Figure 6:**
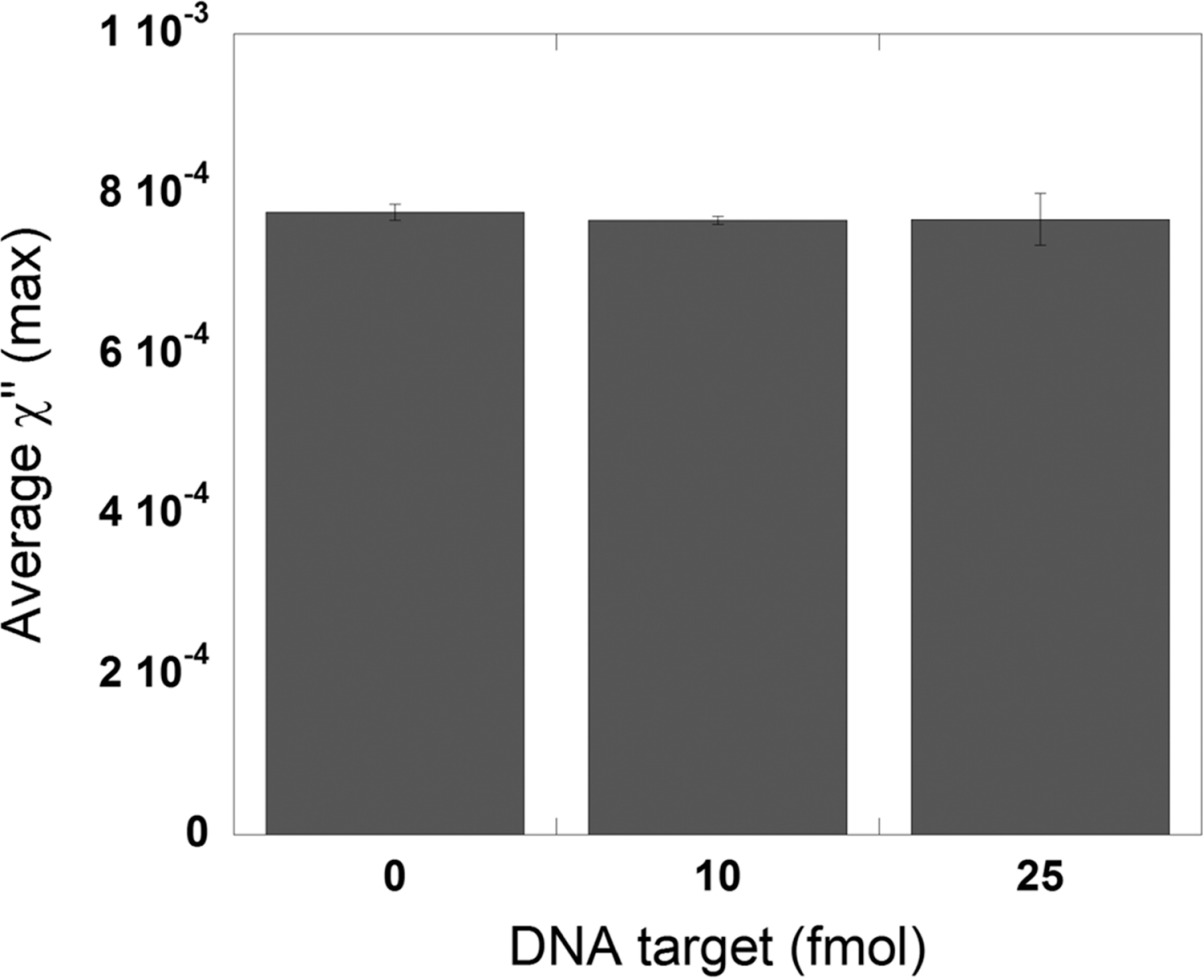
Specificity of the bioassay. Samples with 0, 10, and 25 fmol of starting target were amplified for 20 min of RCA. Magnetic nanoparticles at a concentration of 2 mg/mL functionalized with noncomplementary detection oligos were used for detection. The error bars indicate the standard deviation of three independent replicates.

Specificity is incredibly important in these types of assays, as false positives can lead to improper diagnosis and treatment. The presented assay has two binding steps which are nucleotide sequence-specific. First, the padlock probe only ligates when a complementary target sequence is present. However, if unwanted circularization and amplification occur, the detection oligonucleotides bound to the magnetic nanoparticles would not hybridize with said RCA products. To test the specificity of the RCPs, we functionalized a different detection oligonucleotide to the magnetic nanoparticles, whose sequence does not match that of the RCPs. The results can be seen in Figure 6, and the full imaginary parts of the complex susceptibility spectra for the different samples are presented in Figure S5. A single positive control (PC) sample containing 10 fmol of target is also included in Figure S5 to verify that our samples had RCA products present. When the assay is performed with 10 or 25 fmol of starting DNA target and the magnetic nanoparticles do not have the matching detection oligonucleotide, there is no binding, and the χ″-peak value does not decrease compared to samples with no DNA. A sample of 10 fmol was tested with the correct detection oligonucleotides to show that there was DNA in the sample and had the expected positive response (data not shown). These results show that the assay is specific as there is no RCP–magnetic nanoparticle interaction in all tested samples.

## Conclusions

We have developed a magnetic nanoparticle-based bioassay where target DNA has been amplified through RCA on the surface of microparticles (Dynabeads). We measured the hydrodynamic volume changes in the particles by the change in their Brownian frequency using an AC susceptometer. As the nanoparticles bind, the change is proportional to the size of whatever they bind to. The use of a Dynabead of micrometer size allows for shorter RCA times, as the Dynabead increases the hydrodynamic volume of the bead–RCP complexes, and thus improves the AC susceptometry readouts. A synthetic DNA target sequence was used as a model target in this work. This detection method could be implemented to test other DNA targets, as padlock probes can be designed for other sequences.

The effects of Dynabead concentration, nanoparticle concentration, and RCA time on the performance of the detection assay have been evaluated. It was shown that the optimal conditions for the studied detection method were achieved when using 10 and 1.5 mg/mL Dynabeads and magnetic nanoparticles, respectively. It was also shown that 20 min of RCA was enough time to achieve similar results compared to 40 and 60 min. In addition, an LOD of 0.75 fmol of DNA target was achieved with an RCA time of 20 min. Finally, we describe the specificity of the assay and test whether the magnetic nanoparticles are able to bind to RCPs when the magnetic nanoparticles are functionalized with noncomplementary detection oligonucleotides. The results show that there is no binding up to 25 fmol of starting target.

As a next step, we intend to integrate the molecular reactions to a microfluidic format. This would enhance the degree of automation and reproducibility, which are parameters important to clinical applications.

## Materials and Methods

Sequences of target, padlock probes, and detection oligonucleotides can be found in Table 2.

**Table 2:** Sequences Used in This Study

	sequence (5′-3′)
padlock probe^a^	TGACCCCATTTCCCTGGTGTATGCAGCTCCTCAGTAATAGTGTCTTACGACGCAACTTCACCGAATGAAAGGATATGCTATCYTGAA
target^a^	biotin-CTCTCTCTCTCAGGGAAATGGGGTCATTCARGATAGCATATCCTT
detection oligo^a^	biotin-TTTTTCCTCAGTAATAGTGTCTTAC
detection oligo^b^	biotin-TTTTTTTTTTTTTTTTTTTTGTTGATGTCATGTGTCGCAC

a Sequences used in the optimization work and dose–response curve.

b Sequence used in the specificity experiments.

### Target Recognition, Ligation, and RCA

Target recognition and amplification were performed as follows: 10 μL of target DNA (10 μL of milliQ water in negative NC) was added to a 10 μL ligation mixture for a final concentration of 100 nM phosphorylated padlock probes (biomers.net, Ulm, Germany), 0.2 μg/μL bovine serum albumin (BSA, Thermo Fisher Scientific, Sweden), 250 mU/μL Tth DNA ligase (GeneCraft, Köln, Germany), and 1× Tth ligase buffer. Hybridization and ligation were performed for 5 min at 60 °C. MyOne streptavidin T1 Dynabeads (Thermo Fisher Scientific) were washed three times in 1× Wtw buffer (10 mM Tris-HCl, 5 mM EDTA, 0.1% Tween 20, 0.1 M NaCl). The biotinylated target DNA was captured onto the surface of the washed MyOne Dynabeads (for 10 min), and unreacted padlock probes were removed by washing with 1× Wtw buffer using a permanent magnet. Then, 20 μL of RCA mixture containing 0.2 μg/μL BSA, 125 μM dNTP (Thermo Fisher Scientific), 100 mU/μL phi29 DNA polymerase (Thermo Fisher Scientific), and 1× phi29 buffer was added to the MyOne Dynabeads. RCA was then performed at 37 °C for 10, 20, 40, or 60 min, followed by enzymatic inactivation at 65 °C for 1 min. All samples were made in triplicate.

### Conjugation of Detection Probes to Magnetic Nanoparticles

First, 100 μL of avidin-functionalized magnetic nanoparticles (Micromod Partikeltechnologie GmbH, Rostock, Germany), with a nominal particle diameter of 100 nm and a concentration of 10 mg/mL, was washed three times with 1× Wtw buffer using a permanent magnet. The nanoparticles were resuspended in 1× Wtw buffer in half its volume and incubated with 13 μL of 10 μM biotin-conjugated oligonucleotides (biomers.net) for 30 min at room temperature. After the incubation step, the particles were washed three times with 1× Wtw buffer and resuspended in 1× PBS in its original volume. Finally, the nanoparticle solution was diluted with 1× PBS to the desired particle concentration (0.5, 1, 1.5, or 2 mg/mL, specified for each experiment).

### AC Susceptibility Measurement on Samples Containing Rolling Circle Amplification Product and Probe-Tagged Magnetic Nanoparticles

Equal volumes of RCPs and probe-tagged magnetic nanoparticles were mixed, and the mixture was incubated for 20 min at 60 °C. The frequency-dependent magnetic susceptibility was measured at room temperature using an AC susceptometer (DynoMag, Acreo, Sweden) in the frequency range of 5–25 000 Hz with 17 frequency points. In the case of the specificity experiments, 16 points between 5 and 10 000 Hz were measured. The total measurement time was 20 min. Triplicates of all sample types were measured, and the mean χ″ values and corresponding standard deviation were calculated. In this study, the limit of detection (LOD) was defined as the lowest tested amount of the DNA target yielding a magnetic response that differed at the χ″-peak value by more than three standard deviations from that of the NC.

## Supporting Information Available

Full imaginary part of the complex susceptibility spectra for all experiments (PDF)
