# Supplementary material for: Rolling Circle Amplification on a Bead: Improving the Detection Time for a Magnetic Bioassay
Source: ACS Omega. 2023 Jan 18;8(4):4391–7. doi: 10.1021/acsomega.2c07992 (PMC9893745; doi:10.1021/acsomega.2c07992)
Supplement: Supplementary file 1 — ao2c07992_si_001.pdf [file ao2c07992_si_001.pdf]

SUPPORTING INFORMATION for

Rolling circle amplification on a bead: improving the detection time for a magnetic bioassay

Darío Sánchez Martín[1], Reinier Oropesa-Nuñez[2], Teresa Zardán Gómez de la Torre[1,‡]

[1]Department of Material Sciences and Engineering, Division of Nanotechnology and Functional Materials, Uppsala University, Ångström Laboratory, Box 534, SE-751 21 Uppsala, Sweden

[2]Department of Material Sciences and Engineering, Division of Solid-State Physics, Uppsala University, Ångström Laboratory, Box 534, SE-751 21 Uppsala, Sweden

[‡]Corresponding address: Teresa Zardán Gómez de la Torre, Ångström Laboratory, Box 534, SE-751 21 Uppsala, Sweden, Teresa.Zardan@angstrom.uu.se

Material and Methods

MNPs and Dynabeads were washed as per the Methods section in any other experiment. Both kinds of beads were then functionalized with oligonucleotides in the same way and ratio as previously mentioned in the Methods section. Both kinds of beads were washed thrice and resuspended in PBS at 10 mg/ml. Tubes containing 40 µl of PBS with either 50 µg of Dynabeads or 40 µg of MNPs (equivalent to 20 µl of 2 mg/ml MNPs) were incubated for 20 min at 60 ˚C as was done for normal samples. Readouts were taken immediately after the incubation.

Results

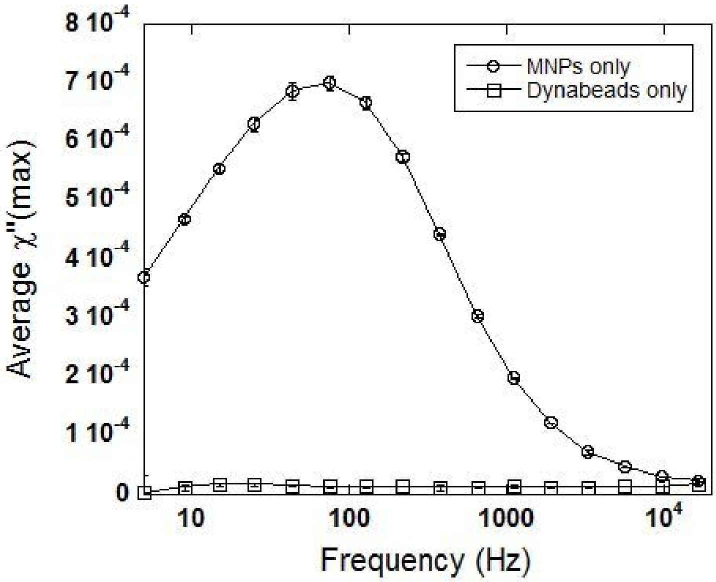

Figure S1. Imaginary part of the complex susceptibility spectra for a sample containing only magnetic nanoparticles and another sample containing only Dynabeads. Error bars represent standard deviation based on triplicates.

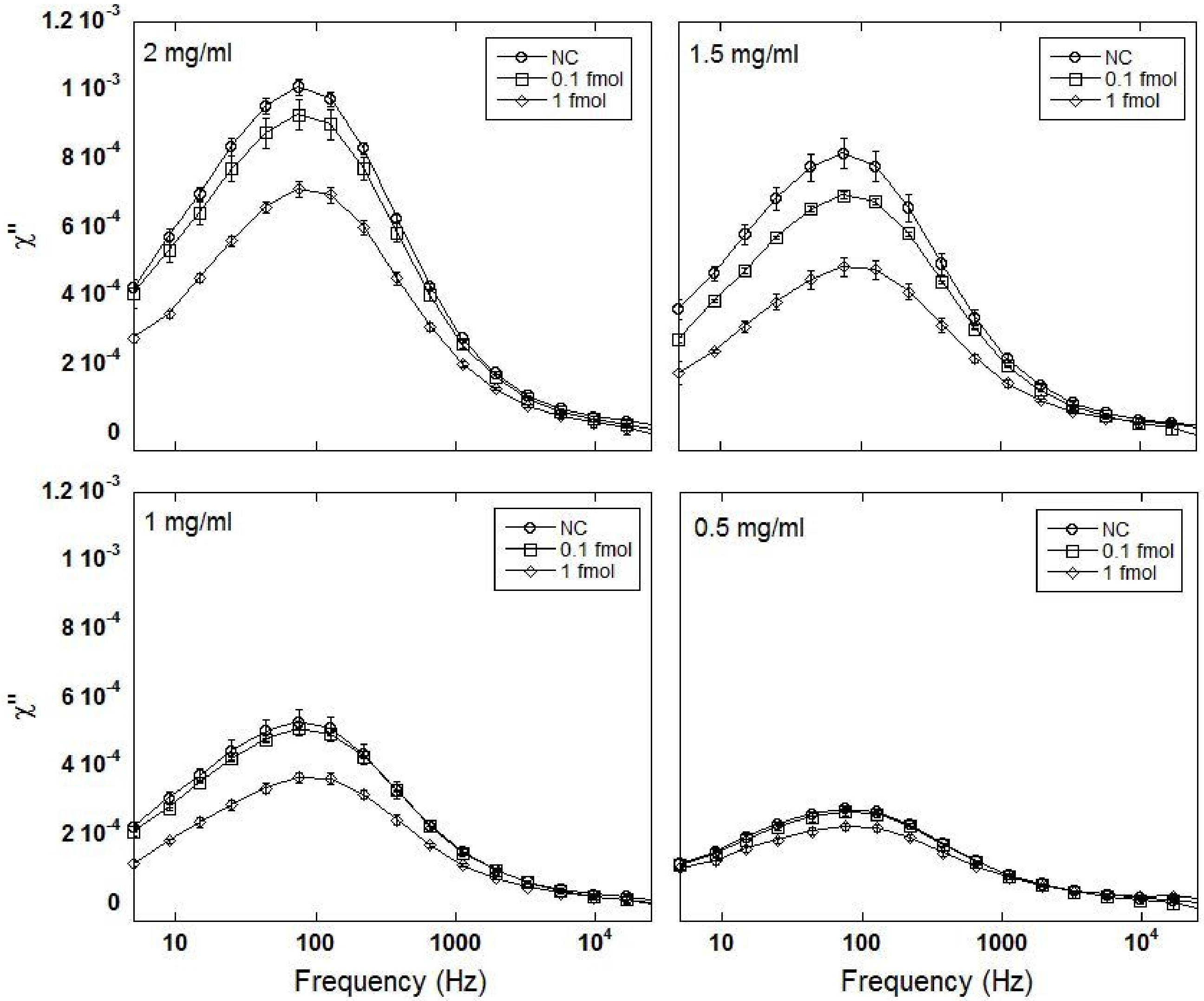

Figure S2. Imaginary part of the complex susceptibility spectra for the experiment presented in Figure 3. Error bars represent standard deviation based on triplicates.

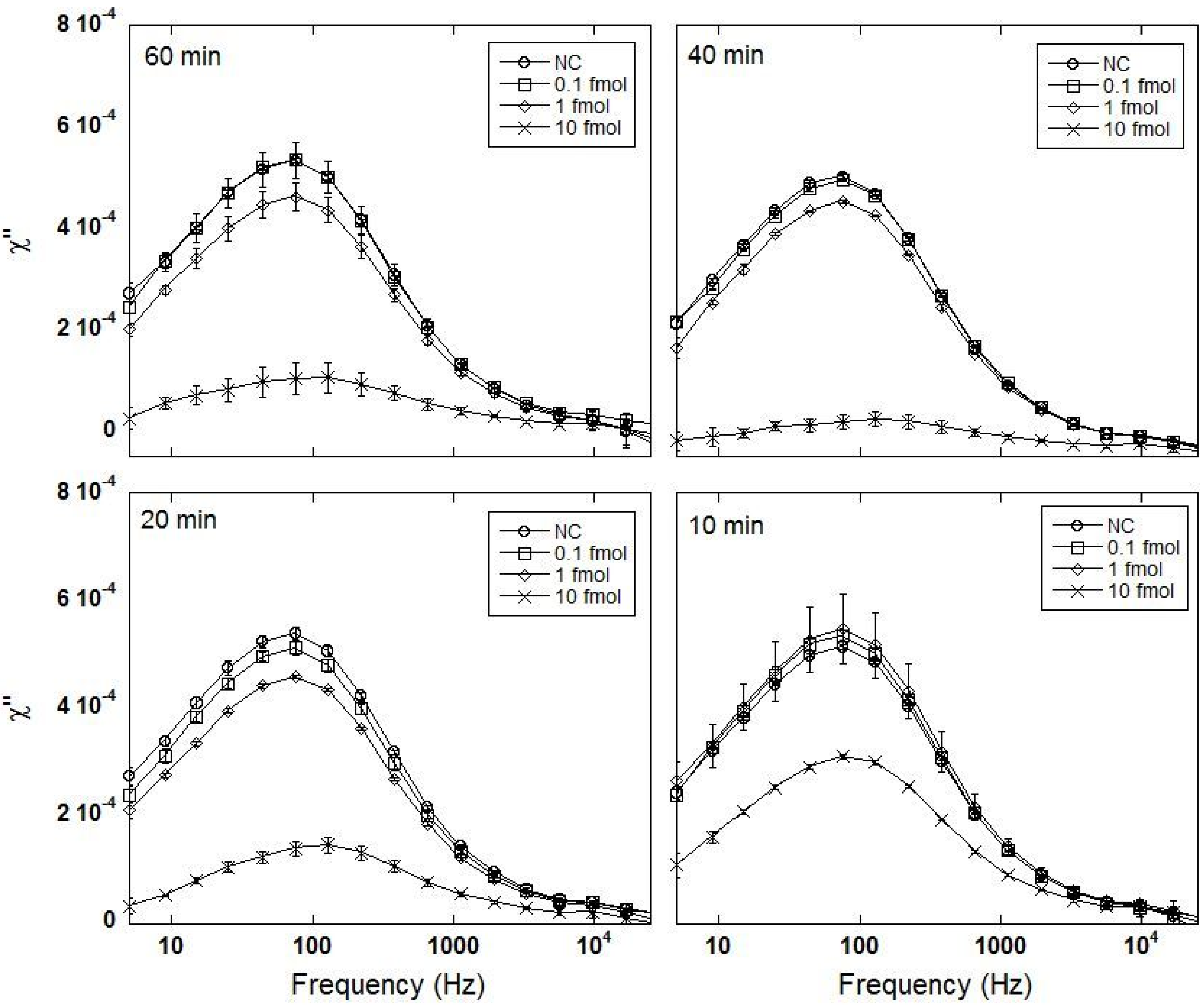

Figure S3. Imaginary part of the complex susceptibility spectra for the experiment presented in Figure 4. Error bars represent standard deviation based on triplicates.

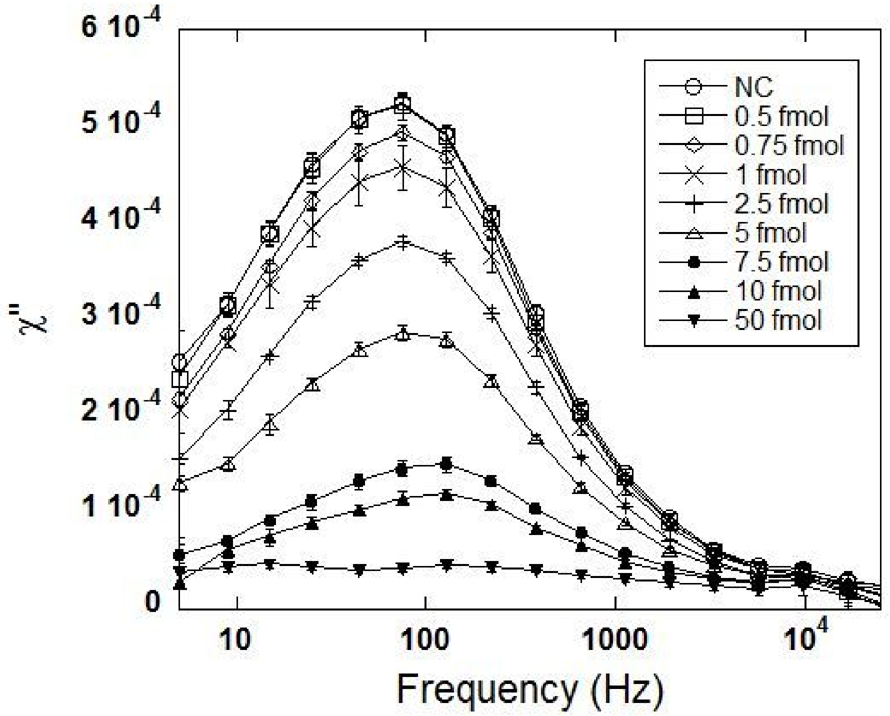

Figure S4. Imaginary part of the complex susceptibility spectra for the experiment presented in Figure 5. Error bars represent standard deviation based on triplicates.

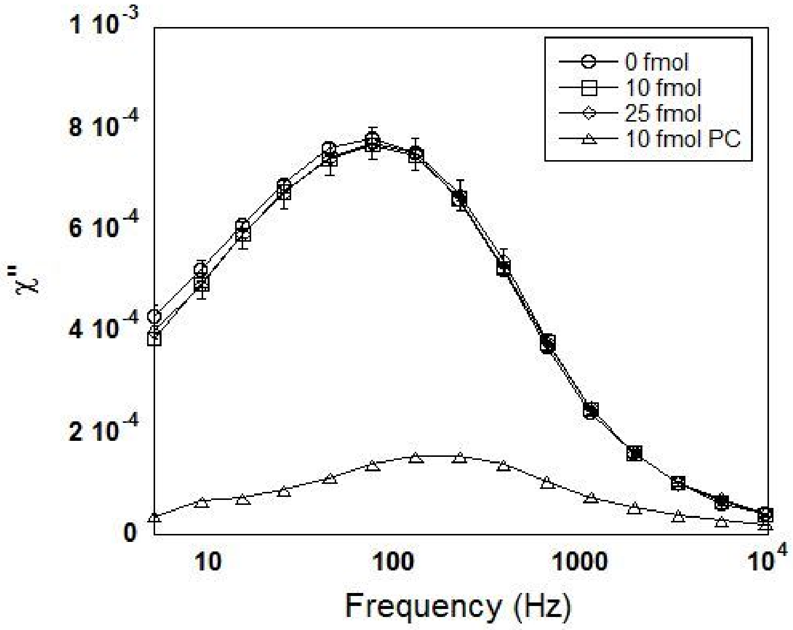

Figure S5. Imaginary part of the complex susceptibility spectra for the experiment presented in Figure 5. Error bars represent standard deviation based on triplicates. A single positive control (PC) sample containing 10 fmol target is also included to verify that our samples had RCA products present.
